# Assessing the reliability and validity of the Risk-Need-Responsivity (RNR) program tool

**DOI:** 10.1186/s40352-022-00182-w

**Published:** 2022-06-09

**Authors:** Niloofar Ramezani, Avi Bhati, Amy Murphy, Douglas Routh, Faye S. Taxman

**Affiliations:** 1 Department of Statistics, School of Computing, George Mason University, 4400 University Drive, MS 4A7, Fairfax, VA 22030 USA; 2 Maxarth, LLC, North Potomac, MD USA; 3 Schar School of Policy and Government, George Mason University, Arlington, VA 22201 USA; 4 University of Utah, Salt Lake City, UT USA

**Keywords:** Risk assessment, Risk-need-responsivity, Reliability, Validity, Fidelity scales

## Abstract

**Background:**

Fidelity assessment tools can assess whether a program embraces a core set of principles and performs well. A quality fidelity tool with valid scales can be a feedback loop to identify areas that need further work to improve the program. Using data collected from 1816 correctional and reentry programs in the United States in the construction sample and 761 programs in the confirmation sample, this study examined the internal consistency of the Risk-Need-Responsivity (RNR) Program Tool, an online resource to capture information about structural features of a program.

**Results:**

The study reports on reliability statistics and factor analyses to highlight individual subscales. Six scales emerged and had acceptable to excellent levels of internal consistency. These scales are staffing, reward-and-sanction, clinical standards for programs, coaching, program duration, and risk-need assessment.

**Conclusions:**

This article discusses fidelity scales from the RNR Program Tool and provides guidance on the importance of tool development processes to ensure accurate, valid, and reliable scales. The purpose of the RNR Program Tool is to create a modern, online tool integrating both the empirical (research) literature on effective practices and clinical standards on quality programming. This process minimizes the need for consultants by giving program administrators the ability to gather information on their programs, score them, and receive instant and targeted feedback with recommendations for improvement to assess their programs against empirical standards in the field. Furthermore, it provides a standardized tool that administrators can use to examine what type of individuals fare better in their programs. The provided targeted feedback can give the programs the ability to seek technical assistance or guidance in specific areas that can strategically strengthen their program.

## Background

Higher quality programs deliver better client-level outcomes (Andrews & Dowden, 2005; Baglivio et al., 2015; Gendreau, 1996; Lowenkamp & Latessa, 2005a, b). Fidelity tools detect how well the program adheres to features of evidence-based practices and treatments (EBPTs) while identifying operational areas where lapses occur (Baglivio et al., 2018; Crites & Taxman, 2013; Hay, 2018; Latessa, 2018; Latessa & Holsinger, 1998). Fidelity tools are also feedback to program administrators on where to make adjustments to better adhere to EBPTs to improve program performance. The domain scores for each EBPT (i.e., use of screening and assessment tools, nature of programming, type of staff, eligibility criteria, etc.) are important to garner the most outcomes from a program fidelity tool. Each individual domain score should be consistent with the underlying principles of EBPTs and clinical practice, and be psychometrically sound.

A significant gap exists in the literature on program fidelity tools. The most frequently used tools are the Correctional Program Assessment Inventory (CPAI; Gendreau & Andrews, 1994), the Correctional Program Checklist (CPC; Latessa et al., 2009; Lowenkamp & Latessa, 2003, 2005a, b), and the Standard Program Evaluation Protocol (SPEP; Howell & Lipsey, 2012; Lipsey et al., 2010). Studies for each tool primarily rely on measuring the overall validity of the tool by using the total score; little attention is given to each specific EBPTs domain scores. Attention to the total score does not describe the program’s adherence to different quality programming features nor reflects performance in a given area. In this study, the RNR Program Tool, which is a relatively new program fidelity tool, is introduced as well as the domains that comport to areas of program performance. This study will (1) apply psychometric principles for domain scales and (2) discuss the importance of psychometrics to scale development. In this paper, we begin with a review of the background about program and program fidelity. Next, we discuss the psychometric methodology of the RNR Program Tool and findings for this instrument. The paper then concludes with a discussion about the importance of psychometrically-sound scales to ensure that the scores are useful for improving program areas.

### Program Fidelity tools in criminal justice

The three prominent program fidelity assessment tools used in criminal justice are: (1) the Correctional Program Assessment Inventory (CPAI), (2) the Correctional Program Checklist (CPC), and (3) the Standardized Program Evaluation Protocol (SPEP). Each tool assesses the quality of the programs but the tools vary in terms of the number and type of item, and sources of information. This section will describe the similarities and differences among the three tools.

#### The correctional program assessment inventory (CPAI)

The CPAI was developed using Canadian programs to measure how well programs were adhering to Gendreau and Andrews’ (1990) principles of effective intervention (Gendreau & Andrews, 1994). The CPAI is a consultant-driven tool where an outside expert collects information via interviews, site visits, and other means on six different domains: 1) program implementation domain focuses on the qualifications and involvement of the program director, the extent to which the program design incorporates the treatment literature, attention to local context such as needs and community values, and the program’s perceived cost-effectiveness (Gendreau & Andrews, 1994; Matthews et al., 2001); 2) assessment procedures refers to risk, needs, and responsivity factors, treatment matching, and treatment quality for clients; 3) characteristics of the program refer to how well the program targets criminogenic attitudes and behaviors and uses incentives and sanctions, addresses treatment modalities and reentry processes to prepare clients for release; 4) staff characteristics identifies training, qualifications, stability, and staffing level of involvement in the program; 5) evaluation regarding the types of feedback and how that feedback is used to monitor program functioning; and 6) miscellaneous category identifies sources of funding and level of community support, and use of ethical guidelines in the program. Each item is measured as a yes/no question with yes responses given one point; the instrument consists of 75 items but 66 items are used to score a program’s overall adherence to core principles. The scores are added together to calculate a total (Gendreau & Andrews, 1994; Matthews et al., 2001). Programs are categorized as: very satisfactory (70–100%), satisfactory (60–69%), needs improvement (50–59%), and unsatisfactory (49% or less).

#### The correctional program checklist (CPC)

The CPC was developed on a sample of halfway house programs in Ohio by researchers at the University of Cincinnati (Lowenkamp & Latessa, 2003, 2005a, b). The CPC can be either a consultant-driven tool or an internal correctional and treatment staff trained as consultants. The CPC and CPAI are similar but have several key distinctions. The University of Cincinnati researchers modified the CPAI by combining the implementation and miscellaneous sections of the CPAI. The CPC has 77 items and five domains that measure the program’s capacity to offer evidenced-based treatments (Duriez et al., 2018; Lowenkamp & Latessa, 2003, 2005a, b). Most CPC items are scored in a similar fashion as the CPAI, using yes/no questions with a yes response receiving one point. Some treatment characteristics and quality assurance domains are weighted and scored on a 0–3-point scale. The scoring categories were modified to include: very high adherence to EBPTs (65–100%), high adherence (55–64%), moderate adherence (46–54%), and low adherence (45% or less). The CPC provides programs with this information along with recommendations for improving their scores.

#### The standardized program evaluation protocol (SPEP)

The SPEP was developed to assess how well juvenile justice programs adhere to the best practice guidelines created from Lipsey’s (2009) meta-analytic review of ‘what works’ for juvenile programs. The SPEP is a self-reported administrator-driven tool. Administrators participate in a training session before completing the SPEP. Twenty-six (26) items cover four domains. Programs are grouped into five categories based on type of program (i.e., restorative, counseling, and skill building), the intensity level, and the comprehensiveness of the program components; more intensive and comprehensive programs receive higher scores (see Lipsey & Chapman, 2017 for more details). The first domain refers to the primary and supplemental program types with two items: (1) the program falls within one of five programming groups (ranging up to 30 points), and (2) whether a qualifying supplemental service is used (measured as yes/no question). The amount of service domain addresses treatment dosage by the percentage of youth that receive the target duration (up to 10 points) and the percentage of youth who receive the target contact hours of the program (up to 10 points). The risk level domain has one item that assesses the percentage of youth with a target risk score set by the juvenile justice system for that program (up to 25 points). Finally, the quality of service domain has 20 questions in four subdomains: protocol, staff training, ongoing staff supervision, and organizational response to drift (five items each). The scoring is out of 100 points where the total number of points defines the program score. The SPEP does not specify scoring categories like the CPAI and CPC (e.g., satisfactory or high adherence to EBPTs).

#### Limitations of the previous program Fidelity tools

The CPAI and CPC share several limitations. First, the scoring is subjective. The determination of whether or not a program receives a point for each item is made by the consultant without rating guidelines or thresholds for meeting criteria on any given item. Second, if there are multiple raters, this can introduce discrepancies in how different areas are rated. While inter-rater reliability (IRR) has been previously established for consultants (see Matthews et al., 2001, for the CPAI), the IRR scores are needed when different internal or external consultants use the tool given the lack of consensus on these items (Holsinger, 1999; Latessa et al., 2009; Lowenkamp, 2004; Lowenkamp & Latessa, 2003, 2005a, b; Makarios et al., 2017; Nesovic, 2003).

The SPEP is a self-administered tool with more clear-cut definitions and guidelines. A limitation for the SPEP is that the tool is only applicable for certain types of juvenile programs. Scoring can be difficult when the meta-analytic database lacks information on a set of items. The lack of a literature base makes it difficult to identify a program group and the appropriate supplemental services, treatment dosage, and risk level to address certain target behaviors. The scoring may under- or over-estimate the true rating of the program.

Table 1 compares three fidelity tools and their respective reliability levels for the main domains and validity. The reliability statistic used by the developers was Cronbach’s alpha, which provides an assessment of the degree to which the items are related to each other, even though most items are dichotomous on the tool. Validity refers to whether it predicts the desired outcome. The reliability of the domains of the CPAI fall short of accepted industry standards. Only the overall CPAI total score is tested for reliability (not the 66 items or the subscale scores). This value suggests that the CPAI does measure program fidelity despite the relatively weak measurement of the intended constructs within the tool (Lowenkamp, 2004).

**Table 1:** Comparison of Program Fidelity Tools in Criminal Justice

	CPAI		CPC		SPEP	
Domain	Reliability	Predicted Validity	Reliability	Predicted Validity	Reliability	Predicted Validity
Program Implementation	.49^d^	.56 + ^d^	unavailable	.41	n/a	n/a
Client Preservice/Offender Assessment	.67^d^	.42 + ^d^	unavailable	.42	n/a	n/a
Characteristics of Program	.43^d^	.52 + ^d^	unavailable	.38	n/a	n/a
Characteristics/Practice of Staff	-.30^d^	.27 + ^d^	unavailable	.55	n/a	n/a
Evaluation/Quality Assurance	.41^d^	.41 + ^d^	unavailable	.16	n/a	n/a
Miscellaneous	-.01^d^	.16^d^	n/a	n/a	n/a	n/a
Primary/Supplement Service Type^b^	n/a	n/a	n/a	n/a	unavailable	−.178
Amount of Service^b^	n/a	n/a	n/a	n/a	unavailable	−.186
Risk Level of Youth^b^	n/a	n/a	n/a	n/a	unavailable	−.42***
Quality of Service^b^	n/a	n/a	n/a	n/a	unavailable	–
Overall	.74	.60 + ^e^	unavailable	.72**^f^	unavailable	−.36***^g^
Number of Items^c^	66	–	77/73	–	26	–

Note: Cronbach’s alpha is used for reliability estimates. Pearson’s r is used for predictive validity estimates. CPAI estimates (Lowenkamp, 2004), CPC estimates (Latessa et al., 2010), and SPEP estimates (Redpath & Brandner, 2010)

+*p* < .10, *p < .05, **p < .01, ***p < .001

^a^CPC’s Leadership and Development domain is the combined Program Implementation and Miscellaneous domains of CPAI

^b^SPEP-specific domains that are similar to CPAI and CPC but use vastly different measures to assess the domains

^c^Number of scoring items only

^d^Estimate for significant items only (see Lowenkamp, 2004)

^e^Predicting return to Ohio Correctional Facility for any reasons (technical violation or new arrest)

^f^Predicting any new misdemeanor or felony conviction

^g^Predicting whether a new complaint was recorded for either delinquency or status offenses

Each of the available instruments does not adequately cover the program fidelity items identified in the literature, as discussed below. The instruments tend to use generic terms such as implementation or quality of service instead of specific constructs or items. The CPC and SPEP do not have any psychometrics published in peer-reviewed journals. And, the literature focuses on the total score outcomes without attention to individual domains. Without the reliability information, or with relatively low reliability, it is difficult to say that the CPAI, CPC, or SPEP can identify program features that are linked to better program outcomes.

The lack of construct validity may be triggering the low levels of reliability seen in the CPAI and potentially the CPC and SPEP. Without establishing construct validity through psychometric approaches, researchers have little way of knowing if their scale is unidimensional or multidimensional (Barchard, 2012; Netemeyer et al., 2011; Raykov & Marcoulides, 2016). The failure to assess the scale items as to which items group together logically and empirically, and to identify items that do not aid in the measurement of a construct need to be addressed. Nesovic (2003) and Lowenkamp (2004) conducted the CPAI’s most recent measurement validity assessment with Nesovic (2003) focusing on face and content validity. Lowenkamp (2004) assessed the reliability of the CPAI’s domains with Cronbach’s alpha without testing for construct validity. The overall reliability of the CPAI was taken to imply a valid tool. This extends to the CPC and the SPEP. Additionally, the CPC lacks measurement validity (Lowenkamp & Latessa, 2005a, b), while no known studies on the validity of the SPEP have been released.^1^

Predictive validity is frequently used for the existing tools. In general, recidivism is the outcome measure of choice, as shown in Table 1 (note: the studies use different definitions and time periods of recidivism). The CPAI predicts return to prison, the CPC predicts any new misdemeanor or felony, and the SPEP predicts general recidivism, or three different outcomes. Studies tend to use the total score to examine the impact on outcomes, and generally programs with higher scores have reduced recidivism outcomes (Holsinger, 1999; Lowenkamp & Latessa, 2003, 2005a, b; Makarios et al., 2017; Nesovic, 2003). But this assumes that the instrument works for various types of programs, and that the type of program is not related to outcomes. And, it assumes that all domains predict the outcomes. For example, Holsinger (1999) and Makarios et al. (2017) included the CPAI or CPC domain scores into models to examine recidivism, which included an effect size for the overall tool score. But neither model discerned whether the assessment tool, implementation, staffing, or other domains had an impact on outcomes.

Both the CPAI and CPC presumes that programming is administered by justice organizations and is primarily cognitive-behavioral.^2^ Such assumptions do not cover the vast majority of programming for justice-involved individuals (see Taxman et al., 2007). Numerous programs may use components of cognitive-behavioral therapy (CBT) in their services, but the bulk of their services may not be rooted in CBT-oriented techniques. A CBT-based framework may be inappropriate for certain types of programs, such as restorative justice and therapeutic communities. Recent advances in the CPC have been to develop variations in the tool for group therapies, drug courts, and community supervision, but it still assumes that CBT is the most relevant programming.

### Measuring program Fidelity

The existing tools do not cover the breadth of justice and/or health programming that individuals may be involved with. Andrews and Bonta (2010) furnished a list that defines the core features of effective programs for the justice system. The core principles recommend to: (1) use human service approaches; (2) use a standardized risk and needs instrument to identify individual areas where improvements are needed; (3) employ behavioral and social learning strategies to help individuals change; (4) tailor services to match the characteristics of individual clients; (5) target programming to multidimensional needs; (6) focus on building strengths of individuals; (7) create a therapeutic milieu; and (8) employ professional discretion to improve client outcomes. These principles are universal regardless of residential, institutional, and community settings where programs are offered. The following summarizes the literature on what we know about each area which provides guidance as to the features that a fidelity instrument should include:

#### Assessments and diagnoses

Quality programming begins with diagnosis, and in the justice arena this includes validated risk and need assessment tools. Validated instruments measure risk for future offending and needs that affect involvement in the justice system (i.e., criminal cognitions and values, peers, substance abuse, employment, etc.). The goals are to use the diagnostic information to improve resource allocation, reduce inconsistencies in decision-making, structure intake interviews to capture similar important information, and improve the matching of individuals to programs (see Singh et al., 2018, for a discussion of different tools; Taxman, 2017; Taxman, 2018). The risk principle identifies an individual’s likelihood of recidivating; the needs principle identifies the individual’s dynamic criminogenic factors that can be targeted by treatment to reduce risk level; and responsivity focuses on matching treatment type, intensity, and duration based on the risk and needs information (Andrews & Bonta, 2010; Crites & Taxman, 2013). Matching clients to levels of services requires attention to individual differences that affect attitude, motivation, and program attendance (Andrews et al., 1990; Gendreau et al., 1996; Lipsey, 2009; Peterson-Badali et al., 2015; Vieira et al., 2009). The type of risk-need assessment tool and how it is used in practice to influence programmatic decisions are important.

#### Clinical programming

Cognitive behavioral therapies (CBT) have been shown to reduce recidivism, and are the preferred style of providing therapeutic services (Cullen & Jonson, 2017; Drake et al., 2009; Tanner-Smith et al., 2012). CBT is effective for a myriad of disorders, including substance abuse, cognitive restructuring, criminal thinking errors, and depression. Other effective approaches are therapeutic communities (National Institute on Drug Abuse, 2014; Sherman et al., 1997), contingency management or the use of incentives for target behaviors (Carroll et al., 2006; Griffith et al., 2000; National Institute on Drug Abuse, 2014; Prendergast et al., 2006; Stitzer & Petry, 2015), social skill development (Sherman et al., 1997), mindfulness therapy (Auty et al., 2017), and interpersonal skill development approaches (Botvin & Griffin, 2004; Landenberger & Lipsey, 2005). For serious opioid or alcohol-related disorders, medication-assisted treatment (Ma et al., 2018), cognitive behavioral therapy (Cullen & Jonson, 2017), and peer navigators (Tracy & Wallace, 2016) have all been found to be effective. Other types of programming that have efficacy are Functional Family Therapy (Robbins et al., 2011) and Multidimensional Family Therapy (Schaub et al., 2014). The type of treatment offered, as well as its programming features, is one key to fidelity.

#### Criminal justice programming

Certain criminal justice programs tend to reduce recidivism such as drug treatment courts (Mitchell et al., 2012) and risk-need-responsivity supervision (Chadwick et al., 2015; Drake, 2011). Similar to clinical programming, higher quality justice programs indicate what services they use, whether they use a curriculum or manual, the staffing to support these programs, and the components of the program.

#### Dosage

Dosage is one of the least studied areas of program fidelity, but the length of a program is important since it defines the opportunity to facilitate behavioral change. Dosage can include the number, frequency, intensity, and duration of treatment services (Crites & Taxman, 2013). Individuals who have been in treatment longer generally experience less recidivism (Bourgon & Armstrong, 2005; Landenberger & Lipsey, 2005; Simpson et al., 1997; Vanderplasschen et al., 2007), and higher risk individuals tend to have better outcomes than lower risk individuals, especially for programs with higher intensity of services (Bourgon & Armstrong, 2005; Kopta et al., 1994; National Institute of Corrections, 2005; Takahashi & Kroner, 2013; Thanner & Taxman, 2003). Examining how the program is delivered in terms of frequency, number of sessions, and length of time details the dosage of the program.

#### Case management

Case management can be defined as the brokerage and matching of services to needs of individuals. Core case management functions are assessment, planning, linking, monitoring, and advocacy (Vanderplasschen et al., 2007). Case managers can instill positive changes such as recidivism reduction, recovery, prosocial thinking and coping skills; they can also address destabilizing factors such as education, employment, health, housing, and transportation.^3^ Proper case management involves quality case plans with identified targeted needs resulting in linking individuals to correct services to support reduced recidivism.

#### Use of rewards and sanctions

Correctional agencies must ensure that individuals comply with court or parole board orders and/or abide by program requirements. The use of rewards and/or sanctions to incentivize or control behavior are compliance tools. Rewards and sanctions have been found to increase rule compliance, which has led to fewer rule violations including drug relapse (Marlowe et al., 2005; Marlowe et al., 2008; Maxwell, 2000; Maxwell & Gray, 2000; McKay, 2017; Robinson et al., 2015). The use of incentives improves compliance more than sanctions (Mowen et al., 2018; Sloas et al., 2019; Wodahl et al., 2011). Graduated sanctions or decision-matrices have aided in appropriately matching sanctions and rewards to levels of compliance (Baglivio et al., 2015; Guastferro & Daigle, 2012; Schumacher & Kurz, 2000). Justice-involved individuals with sanctions appropriately matched to the offense exhibited lower recidivism rates compared to those who received a sanction that was not appropriate (Baglivio et al., 2015; Schumacher & Kurz, 2000). Fidelity tools should assess the range of sanctions and incentives used for different types of behaviors, and the frequency of application.

#### Drug testing

Drug testing is frequently used to gauge compliance to program conditions for drug-involved individuals. Drug testing can be used as a program requirement to monitor behavior, or it can be used as a sanction. Either a random testing schedule or a set schedule is recommended for drug courts (Carey et al., 2008), although studies have not confirmed which one produces better outcomes. The manner in which drug testing is employed in a program in terms of its frequency and the responses to positive or negative tests can be assessed for adherence to quality programming.

#### Clinical standards

Delivering clinically-related programs with integrity has been shown to have an effect on recidivism. Features of a clinical program are client’s capabilities to be in the program (MacKenzie, 2000), use of a manualized treatment program (Fixsen et al., 2005; Fixsen et al., 2015; Howell & Lipsey, 2012; Mann, 2009), staff with appropriate credentials (i.e., Masters level, Ph.D.) and certification(s) and staff trained in the type of therapy they are responsible for delivering (Simons et al., 2010; Stanard, 1999). Each is a marker of quality programming.

#### Quality assurance

Many agencies use a variety of policies and procedures to manage the quality of their programs, which have been shown to improve program outcomes (Magnuson et al., 2019; Rudes et al., 2013). Procedures vary including evaluations of the program, external audits of the program, internal quality reviews, supervisor or management review of the cases, staff peer review, and the coaching of staff. Fidelity tools should include measures of quality assurance processes which vary in respect to their procedures – and the frequency with which those procedures are used.

## Methodology

The purpose of this study is three-fold: (1) to psychometrically evaluate the scales of a new fidelity tool, the RNR Program Tool, (2) to utilize psychometric scale development to design the existing scales and improve their internal consistency, and (3) to provide guidance on the importance of psychometrics in tool and scale development. In addition, we report the findings on two sample sets of programs data to illustrate how the scores vary across types of programs. This study is intended to provide program administrators with knowledge about fidelity of their programs. Using data collected from 1816 correctional and reentry programs in the United States in the construction sample and 761 programs in the confirmation sample, this study examined the internal consistency of the RNR Program Tool. Exploratory factor analysis (EFA) and reliability analyses were performed on the construction sample to let the data determine underlying constructs for the measured variables. Then, confirmatory factor analysis (CFA) was performed on a different sample to test and confirm the structure of the identified factors. More details are explained below.

The RNR Program Tool was developed in 2009–2011 to provide a self-administered instrument for a broader array of programming that is typically used for justice-involved individuals including cognitive behavioral groups, restorative justice, educational groups, and community services; the tool was designed to assess the fidelity of program components depending on the goal, purpose, and whether the program had a therapeutic orientation(s). The RNR Program Tool includes a number of built-in reliability checks. It does not require consultants to administer it but instead allows agencies and systems to use the tool to broadly examine the quality of their programs and services. Training and certification for the tool are provided by the Center for Advancing Correctional Excellence (ACE!) (see Taxman & Pattavina, 2013).

### Sample

Data were collected from programs across the United States that agreed to use the Program Tool as part of the RNR Simulation Tool package (www.gmuace.org/tools). Many of the programs were newly awarded grants from the Center for Substance Abuse Treatment (U.S. Department of Health and Human Services) and/or Bureau of Justice Assistance (U.S. Department of Justice). Both granting agencies encouraged their grantees to use the tool to assess the quality of the program. Others are from select jurisdictions that requested the assistance of the ACE! to improve program fidelity. Program staffers completed the tool and agreed to allow the information to be used for research purposes. The data included a construction sample of 1816 correctional and reentry treatment programs in the construction sample and 761 programs in the confirmation sample. The tool involves a self-administered online survey that is stored in a secure database.

The Program Tool asks questions about the treatment programs, program characteristics, and demographics of individuals served. This included information about the client population the program serves; the program staff, capacity, completion rates, and funding sources; program operations and performance measures, including operation protocols, participant service screening, referrals, offerings, dosages, and processes; and program implementation factors such as training, evaluations, and barriers. A comprehensive description of the RNR Program Tool is detailed in Taxman and Pattavina (2013).

### Analytic plan

Various techniques guided the scale development for the RNR Program Tool and assessment of internal consistency. First, EFA and reliability analyses were performed on this sample using Stata 16 (StataCorp, 2019). EFA was used in this study to determine underlying constructs for the measured variables. Considering EFA and CFA are complementary methods, after conducting EFA to allow the data identify a scale’s underlying latent constructs, CFA was performed on a different sample by using MPLUS version 8.6 (Muthén & Muthén, 2017) to verify the factor structure of the observed variables and confirm the consistency between the scale and theoretical structure (Capik & Gozum, 2011; Tabachnick & Fidell, 2001). So, the second phase of the study applied CFA to a confirmation sample to test the assumption that the RNR program tool measures the latent constructs defined by EFA and based on expert opinion. The Kuder Richardson, an equivalent of Cronbach alpha statistic for binary items, was used to guide the inclusion and exclusion of items and assess internal consistency of the scales.

In the EFA, the eigenvalues, scree plot, and theoretical basis of the program risk, need, and responsivity were considered to select the optimal number of factors. After EFA was conducted with an oblique PROMAX rotation, CFA was performed using diagonally weighted least squares—mean and variance adjusted (WLSMV) estimator. The data were examined based on correlations among variables, parameter estimates, and the model’s fit to the observable data. Model fit was assessed by examining the relative chi-square (chi-square/degrees of freedom), root mean square error of approximation (RMSEA), probability RMSEA, standardized root mean square residual (SRMR), and comparative fit index (CFI) and Tucker Lewis index (TLI). Factor loadings of each item were expected to be 0.40 or greater (Nunnally, 1978). Large values of chi-square test of model fit is expected for large sample sizes (Brown, 2015); therefore, is rarely used in applied research as a sole index of model fit, yet reported here due to common practice. RMSEA is a widely used and recommended index under the category of parsimony correction fit indicis (Steiger & Lind, 1980) and RMSEA values of 0.01, 0.05 and 0.08 indicate excellent, good, and mediocre fit, respectively, while some go up to 0.10 for mediocre. Even though this index is in nature different from other absolute fit indicis, Hu and Bentler (1999) argued that they could be grouped under the category of absolute fit and used when it is difficult to measure absolute fit using other measures such as SRMR and χ2. Probability RMSEA <= .05 indicates a good fit adjusting for model parsimony. SRMR values less than .08 are indicative of good fit. As explored by Shi et al. (2020), for binary data, under varying conditions, the SRMR values are consistently overestimated within CFA, suggesting that the model fits poorer than what it actually does. CFI values can range between 0 and 1, with values greater than 0.90 indicating good fit. TLI ranges between 0 and 1, with values greater than 0.90 indicating good comparative or incremental fit (Brown, 2015). Considering that the nature of our data is binary, the fact that we have a large sample size and a relatively large number of items and factors, the affirmentioned literature suggest focusing on RMSE, CFI, and TLI to assess the collective goodness-of-fit.

Finally, each of the subscales was expected to have reliability with a Kuder-Richardson of 0.60 or greater (Hulin et al., 2001).

#### Reliability: Cronbach’s alpha and Kuder-Richardson

The reliability of an instrument or questionnaire is concerned with the consistency, stability, and dependability of the scores (Hancock et al., 2010). Reliability relates to the consistency of the scores measured within an instrument. The less consistency that exists within a given measurement, the less useful the data may be in specifying a construct. While Cronbach’s (1951) alpha is the most commonly used reliability estimates (Hogan et al., 2000; Kaplan, 2004), it is best suited for evaluating items scored in multiple answer categories. When estimating internal consistency for dichotomously scored items, the *Kuder Richardson Formula 20* (known as KR-20 formula) is recommended. KR-20 formula is equivalent to performing the split half methodology on all combinations of questions and is applicable when each question is either right or wrong (dichotomous). KR-20 coefficient was measured and reported below.

#### Validity, exploratory factor analysis, and confirmatory factor analysis

As noted above, EFA is considered a variable reduction technique for correlated items or variables. A factor analysis approach to data reduction is a fundamentally different technique than other variable selection techniques, such as principal component analysis, since it measures latent variables to examine the construct validity and psychometric properties of an instrument (Yu & Richardson, 2014). Since this study required the identification of underlying latent constructs to build scales and assess construct validity, EFA was performed on the scales of the RNR Program Tool. After conducting the EFA to develop the latent constructs, CFA was run on a new round of collected data using 34 items for measurement of model, to confirm the factor structure and dimensionality.

## Results

### Internal consistency and score development

**Fig. 1:**
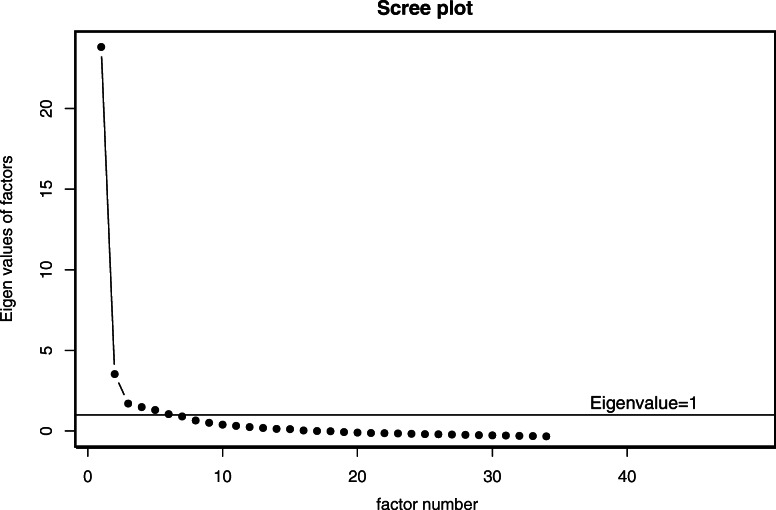
Scree Plot from EFA (*N* = 1816)

The Risk-Need-Responsivity (RNR) Program Tool scales established construct validity using EFA and CFA, and had good reliability, with KR-20 reliability coefficients of 0.60 to 0.90. All items were dichotomized to ensure the consistency of the items. The reliability measures can be found in Table 2. EFA was conducted on the items with an oblique PROMAX rotation, which examined the dimensionality of each construct by determining the existence of relationships between factors when the information of the dimensionality was limited (Netemeyer et al., 2003). EFA identified six factors with eigenvalues above one, which was set as the cutoff point for these values. Figure 1, scree plot from EFA, shows that factor analysis suggested six factors. The horizontal line on this figure shows the eigenvalue of one, which was used as the cutoff point. Coupled with risk-need-responsivity theory and how data loaded into different factors within the EFA analysis, we identified six meaningful factors. Beside these six factors, three other constructs with few items and lower factor loadings (i.e. drug testing, responsivity, and quality assurance) originally emerged but had low reliability. Since modification efforts to boost their reliability were unsuccessful and they were posing cross loading and other issues, they were dropped.

**Table 2:** EFA and Reliability Results

Factor	#Items	Eigenvalue from EFA	KR-20 reliability coefficient for main sample (*N* = 1816)	KR-20 reliability coefficient for confirmation sample (*N* = 761)
Staffing (1)	8	17.277	.90	.75
Reward/Sanction (2)	9	4.237	.85	.80
Clinical Standards (3)	4	2.052	.71	.64
Coaching (4)	4	1.493	.60	.60
Program Duration (5)	4	1.335	.63	.64
Risk-Need Assessment (6)	5	1.095	.86	.87
Total Score	34		.91	.90

Table 2 shows how many items were loaded in each factor, the eigenvalues, and reliability measures for these six factors with eigenvalues greater than one. In the factor analysis, the first factor will account for the most variance, the second will account for the next highest amount of variance, and so on. The fit statistics of the EFA showed good fit (RMSEA = .039, CFI==.988, TLI = .981, SRMR = .045). Chi-square value cannot be considered as a reliable measure of fit for this analysis with the sample size of 1816; as discussed in Brown (2015), χ2 is inflated by sample size for sample sizes of 100 and above and so is rarely used in applied research as a sole index of model fit, especially in the presence of non-normal data and higher sample sizes (both present in this study).

Internal consistency across almost all factors was high. With the KR-20 coefficient, a score above .5 is usually considered reasonable. Good internal consistency means that the survey items tend to pull together. In other words, a participant who answers a survey item positively is more likely to answer other items in the survey positively (Blunch, 2008).

The staffing factor consists of 8 items with a high reliability KR-20 coefficient of .90. The reward-and-sanction factor consists of 9 items with a high reliability KR-20 coefficient of .85. Four items loaded into factor 3, clinical standards for programs, with a reliability KR-20 coefficient of .71. Four items loaded together to create a coaching factor (4) with a reliability KR-20 coefficient at .60. Factor 5 has four items representing the program duration with a reliability coefficient of .63. Five items loaded together to create the use of risk-need tools factor with a reliability measure of .86 (factor 6). Total score has a reliability of .91 for the main sample of 1816 individuals and a comparable reliability of .90 for the test sample of 761, which was used for the CFA. Originally, we had expected to have 3 more factors (quality assurance, responsivity and use of drug test), but they had low reliability, very few items with cross-factor loadings, and the CFA confirmed excluding them. Therefore, number of factors was reconsidered because very few items are insufficient to measure a construct (Norton, 1983) and items loading on more than one factor are suspect (Wood et al., 2015).

### Confirmatory factor analysis

CFA was conducted on the items on a different sample of 761 programs to test the hypotheses about the six theorized constructs achieved by EFA, which concurred with expert opinions and theoretical concerns. WLSMV estimator was used within CFA, which is specifically designed for binary, categorical, and ordinal data. As Li (2016) shows, WLSMV is less biased and more accurate than robust maximum likelihood method in estimating the factor loadings across nearly every condition in the presence of non-continuous items (binary items in this study).

#### Model fit

Model fit was assessed and the results were satisfactory; the collective goodness-of-fit indices pointed to a good fit. Chi-Square Test of Model Fit was 1463.094 with 512 degrees of freedom. Just like the EFA, a large Chi-square value is expected for large sample sizes (Brown, 2015); therefore, not a good measure of fit for non-normal data and higher sample sizes. While χ2 is routinely reported in CFA research, other fit indices (e.g., the Tucker–Lewis index) are recommended (Brown, 2015) and used here. RMSEA, which is a widely used and recommended index under the category of parsimony correction fit indicis (Steiger & Lind, 1980), was estimated to be 0.049, which indicates a good to excellent fit.^4^ Another measure of model fit is probability RMSEA <= .05, which was 0.631, indicating a good fit adjusting for model parsimony (Brown, 2015).^5^ CFI value of .937 showed a good fit^6^ as well as TLI, which was.931^7^ (Brown, 2015). Finally, SRMR was .105; although this value is greater than .08, considering the other fit indices, we believe we have an overall good fit. As explored by Shi et al. (2020), for binary data, the SRMR values are consistently overestimated within CFA and is not a good measure of the “close” fit.

**Fig. 2:**
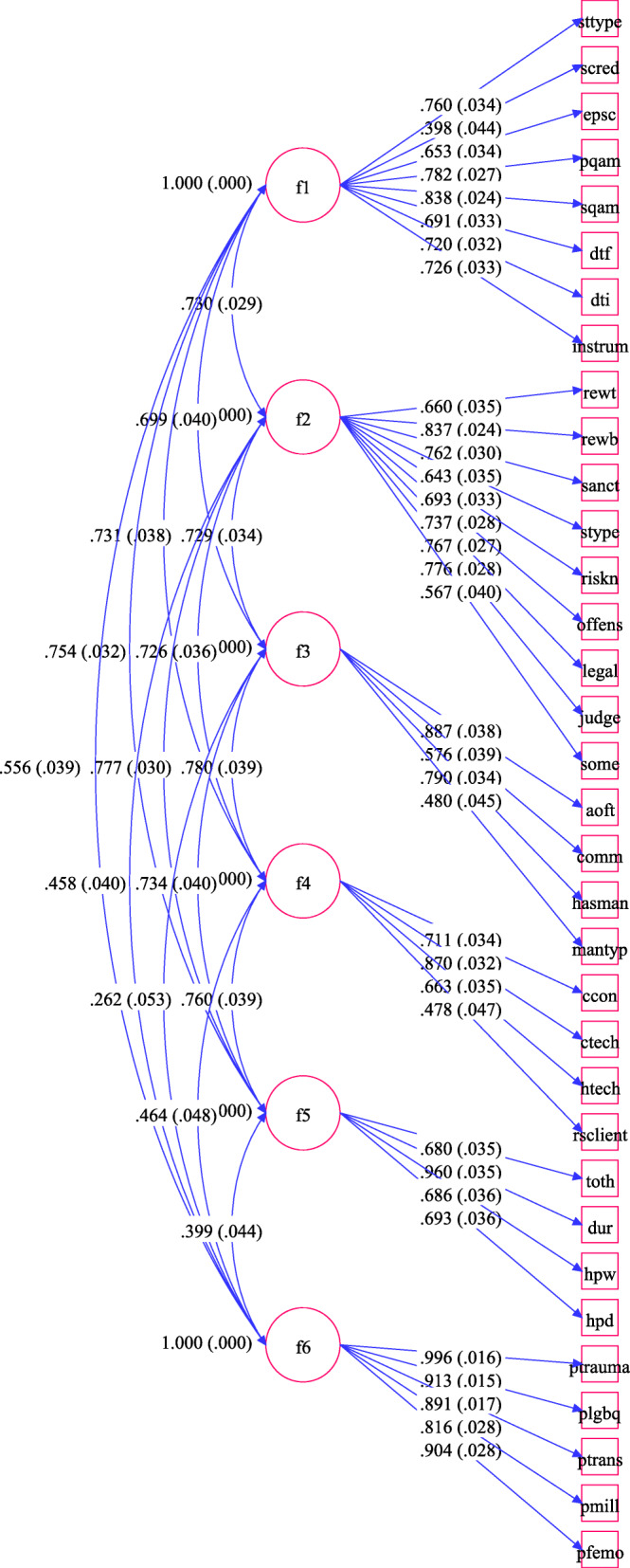
CFA Diagram

Table 3 shows the factor structure, the standardized parameter estimates, standard errors and significance (*p*-value) for the loadings of all six latent variables of the RNR scales. Figure 2 shows the CFA diagram, which includes all the parameter estimates, standard errors, and correlations, including correlations among the six latent constructs (i.e. F1 through F6). Suggested modifications based on modification indicies were explored; however, the gain in terms of model improvement was minimal. According to Brown (2015), several problems may occur when models are re-specified solely on the basis of modification indices or standardized residuals; therefore, letting data-driven modifications alone decide for the model may negatively affect the generalizability of the results. Consequently, considering that these modifications were not supported, neither they could be justified, on the basis of prior theory and we already had an overall good fit, in order to avoid overfitting (i.e., adding unnecessary parameters to the model), we did not re-specify the model (MacCallum et al., 1992).

**Table 3:** CFA Model Results

	Item description	Item	Estimate	S.E.	Est./S.E.	*P*-Value
F1	Staffing	BY				
	Staff Type	STTYPE	0.760	0.034	22.382	<.0001
	Staff Credential Scale	SCRED	0.398	0.044	8.976	<.0001
	Evaluation Performed Scale	EPSC	0.653	0.034	19.063	<.0001
	Primary Quality Assurance Measures	PQAM	0.782	0.027	29.095	<.0001
	Secondary Evaluation Scale	SQAM	0.838	0.024	34.757	<.0001
	Drug Test Frequency	DTF	0.691	0.033	20.646	<.0001
	Drug Test Inconclusive	DTI	0.720	0.032	22.172	<.0001
	Target Specific Assessment	INSTRUM	0.726	0.033	21.758	<.0001
F2	Reward Sanction	BY				
	Rewards Used	REWT	0.660	0.035	18.847	<.0001
	Reward Process	REWB	0.837	0.024	35.219	<.0001
	Sanctions Used	SANCT	0.762	0.030	25.123	<.0001
	Sanction Methods Scale	STYPE	0.643	0.035	18.533	<.0001
	Risk/need Assessment	RISKN	0.693	0.033	20.732	<.0001
	Current/past Offense	OFFENS	0.737	0.028	25.943	<.0001
	Legal Status (parole/prob./etc.)	LEGAL	0.767	0.027	27.994	<.0001
	Clinical/professional Judgement	JUDGE	0.776	0.028	28.107	<.0001
	Court Mandates	SOME	0.567	0.040	14.237	<.0001
F3	Clinical Standards	BY				
	Frequency of Programming	AOFT	0.887	0.038	23.329	<.0001
	Com Type	COMM	0.576	0.039	14.765	<.0001
	Has Manual for Treatment	HASMAN	0.790	0.034	23.423	<.0001
	Includes Worksheets	MANTYP	0.480	0.045	10.749	<.0001
F4	Coaching	BY				
	Client Contact Types Scale	CCON	0.711	0.034	21.128	<.0001
	Coaching Techniques Scale	CTECH	0.870	0.032	26.797	<.0001
	Has Tech	HTECH	0.663	0.035	18.799	<.0001
	Refer Services to Client	RSCLIENT	0.478	0.047	10.242	<.0001
F5	Program Duration	BY				
	Total Hours	TOTH	0.680	0.035	19.419	<.0001
	Duration	DUR	0.960	0.035	27.778	<.0001
	Hours per Week	HPW	0.686	0.036	19.155	<.0001
	Has Phase Duration	HPD	0.693	0.036	19.445	<.0001
F6	Risk Need	BY				
	Population Treated for Trauma	PTRAUMA	0.996	0.016	62.680	<.0001
	Population is LGBQ	PLGBQ	0.913	0.015	59.634	<.0001
	Population is Transgender	PTRANS	0.891	0.017	51.395	<.0001
	Population Uses Mindfulness	PMILL	0.816	0.028	28.999	<.0001
	Population Female Offender	PFEMO	0.904	0.028	32.397	<.0001

## Discussion

Program fidelity is associated with better outcomes, and it is important to assist correctional and treatment organizations – either institutional or community-based – with the tools to understand the degree to which they are implementing quality programs to achieve key client benchmarks (Andrews & Dowden, 2005; Baglivio et al., 2015; Gendreau, 1996; Lowenkamp & Latessa, 2005a, b). Fidelity assessment tools were developed as a means to provide a structured, objective method for both scoring the program’s implementation and providing feedback on areas where programs can be strengthened. For the program fidelity tool to be useful, it must have valid scales appropriate for that program type. The three most common instruments in the field – CPAI, CPC, and SPEP – have not consistently provided the psychometric foundation for their subscales or total scores.

In this study, we used sound psychometric principles to examine the construct validity and reliability of domains for the RNR Program Tool for both a construction and validation sample of programs. Assessing and establishing construct validity is a critical step to ensure that the measures used are actually measuring the desired construct. From this study we were able to construct scales for staffing, use of rewards and sanctions, clinical standards, coaching, program duration, and risk-need assessment—all of which are recognized as important factors to deliver high caliber programs and services by either correctional, treatment, or general service providers. We were not able construct measures for drug testing, responsivity, and quality assurance because theses scales were not independent from the seven domains that were created. Using valid construction and validation methods and solid psychometric procedures, we were able to identify the items necessary to accurately measure each construct, which ranges from four items (clinical standards, coaching, and program duration), five items for risk-need assessment, eight items for staffing, to nine items for reward and sanctions. Psychometric methods aid researchers to assess if the construct is unidimensional (measuring only one construct) or multidimensional (measuring more than one construct). Collectively, the advancement of rigous scales is an important contribution to the field.

Future research is needed to examine the predictive validity for different program outcomes (e.g., recidivism reduction, infraction reduction, successful program completion, or adherence to program guidelines) for each domain and the overall tool score. Predictive validity will examine which of the domains are needed to deliver a high-quality program that contributes to positive client outcomes. We could not perform concurrent validity for this study because there are no other validated instruments that are related to the constructs measured in this study, and we are unaware of any tools that have such items. In another study, we explored how program- and individual-level factors impact the success of 848 drug court clients in nine courts in terms of: 1) graduation rates; and 2) not being arrested while participating in the court program. In this study, we found that three program level factors (staffing, rewards-and-sanctions, and program duration) predicted recidivism (whereas the total score) (Breno et al., 2022). Given that the drug courts were homogenous in their features, it is not suprising that other program features were not related with client outcomes. Further research is needed to identify the core program features that generate client outcomes.

Similar to SPEP, the RNR Program Tool is a self-administered tool that can be completed by administrators. It is offered online, has close-ended questions, and generates a feedback report to the administrator based on their responses to each area. The construct validity and reliability of the scales in the RNR Program Tool offer correctional, treatment, life skills, and educational programs the program tool to use in order to assess whether their own program is structurally sounds. The purpose of the RNR Program Tool was to create rigorous scales built into an online tool that integrates both the empirical (research) literature on effective practices and clinical standards on quality programming. Administrators can gather information on their programs, score them, and receive instant and targeted feedback with recommendations for improvement. The targeted feedback can give the programs the ability to seek technical assistance or guidance in specific areas that can strategically strengthen their program. The tool provides an opportunity for administrators to learn aobut their operations and work on specific areas. While the tool has utility for administrators, consultants can also use the tool to provide a solid process for assessing program features. Facilitators or consultants are frequently needed for an agency to critically analyze operations, and in fact external facilitators typically help organizations achieve greater gains (Berta et al., 2015; Harvey et al., 2018; Lessard et al., 2015; Magnuson et al., 2019). In some ways the design of this particular tool advances fidelity tools by providing robust scales and a means to provide structured feedback to guide program improvements.

Second, RNR Program Tool scales essentially describe core functions that are important to the program quality in terms of the clinical, management, and/or empirical literatures. And, these core features derive from a myriad of literature in correctional programs, substance use disorders, mental health disorders, educational programs, and social work services. This means that the tool and the resulting scale are valuable to different audiences including programs and services that are operated by other agencies outside of the correctional system. These include practices related to staffing, clinical standards, and use of rewards and sanctions. The scales disentangle the messiness of implementation fidelity into more manageable and targeted components given the goal is to provide administrators with feedback on how to improve their current program. That is, the feedback report will generate scores in each domain and then administrators can use this report to identify program features that need to be strengthened. Targeted feedback reports can be directive and point programs into specific directions for improving their fidelity. This self-administered approaches uses feedback reports as a means of helping administrators identify the aspect of the program that needs improvement.

This study illustrates the importance of using accepted psychometrics in developing instruments for justice settings and/or programs that service justice clients. During the development of the RNR Program Tool, EFA and CFA assisted in clarifying the scales. It raised issues regarding the measurement of core variables, which resulted in harmonizing the underlying data to use dichotomous variables. This drove the selection of the KR-20 over the Cronbach’s alpha for measuring reliability. Developers of scales can learn lessons about creating subscales or domains that are empirically and theoretically sound through the use of better psychometric methods. This is important to both the science behind the instrument as well as its utility in the field. More emphasis should be placed on ensuring that instruments are reliable and valid.

## Conclusions

This study is motivated by the dearth of studies on scales for fidelity tools. Fidelity assessment tools can assess whether a program embraces a core set of principles and performs well. After covering the literature on what we know about fidelity in programming and program impact on client level outcomes, this article discusses the construction of scales for one fidelity tool, the RNR Program Tool, which integrates both research on effective practices and clinical standards on quality programming. This online process gives administrators and consultants the ability to collect information on their programs, score them, and receive instant and targeted feedback with suggestions for improvement in their programs and to evaluate them against empirical standards in the field.

The importance of developing accurate, valid, and reliable scales while developing tools is highlighted in this study. A valid fidelity tool is invaluable since it ensures that the underlying scales represent objective items that are useful in assessing the process of a program. Good quality scales can provide a feedback loop to administrators on how to strengthen the program. Consequently, a quality fidelity tool with valid scales can offer an accurate picture of the functionality of a program with guidance on how to improve practice. Furthermore, it provides a standardized tool that administrators can use to examine what type of individuals fare better in their programs. The provided targeted feedback can give the programs the ability to seek technical assistance or guidance in specific areas that can strategically strengthen their program.

## Data Availability

The datasets during and/or analyzed during the current study available from the corresponding author on reasonable request.

## Footnotes

1 The CPC was modeled after the CPAI and the Principles of Effective Intervention (see Gendreau, 1996).

2 More effective treatments are cognitive-behavioral in nature (see e.g., Andrews et al., 1990; Drake et al., 2009).

3 This definition has been adapted from Kanter’s (1989) definition for psychological case management to define case management in a criminal justice context.

4 RMSEA values of 0.01, 0.05 and 0.08 indicate excellent, good and mediocre fit respectively, while some go up to 0.10 for mediocre

5 Even though probability RMSEA <= .05 is in nature different from other absolute fit indicis, Hu and Bentler (1999) argued that they could be grouped under the category of absolute fit and used when it is difficult to measure absolute fit using other measures such as SRMR and χ2.

6 CFI values can range between 0 and 1, with values greater than 0.90 indicating good fit.

7 TLI also ranges between 0 and 1 with values greater than .90 indicating good comparative or incremental fit.
