# Correction: Assessing the reliability and validity of the Risk-Need-Responsivity (RNR) program tool

**DOI:** 10.1186/s40352-022-00186-6

**Published:** 2022-07-07

**Authors:** Niloofar Ramezani, Avi Bhati, Amy Murphy, Douglas Routh, Faye S. Taxman

**Affiliations:** 1 Department of Statistics, School of Computing, George Mason University, 4400 University Drive, MS 4A7, Fairfax, VA 22030 USA; 2 Maxarth, LLC, North Potomac, MD USA; 3 Schar School of Policy and Government, George Mason University, Arlington, VA 22201 USA; 4 University of Utah, Salt Lake City, UT USA

**Correction to: Health Justice 10, 19 (2022)**

**https://doi.org/10.1186/s40352-022-00182-w**

Following the publication of the original article (Ramezani et al., 2022) the corresponding author reported a mistake in the last author’s affiliation. Dr. Faye S. Taxman should only be affiliated to “^3^Schar School of Policy and Government, George Mason University, Arlington, VA 22201, USA” rather than affiliated to both affiliations 1 and 3. The original article (Ramezani et al., 2022) has been updated.
